# Something Got Your Tongue? A Unique Cause of Hypoglossal Nerve Palsy

**DOI:** 10.1155/2022/2884145

**Published:** 2022-11-22

**Authors:** Alan Tesson, Peter Kranz, Ali Zomorodi, Joel Morgenlander

**Affiliations:** ^1^Inova Neuroscience Service Line, Inova Health System, 8081 Innovation Park Dr, Fairfax, VA 22031, USA; ^2^Department of Radiology, Duke University Medical Center, 2301 Erwin Rd, Durham, NC 27710, USA; ^3^Department of Neurosurgery, Duke University Medical Center, 2301 Erwin Rd, Durham, NC 27710, USA; ^4^Department of Neurology, Duke University Medical Center, 2301 Erwin Rd, Durham, NC 27710, USA

## Abstract

**Introduction:**

The authors report a rare cause of isolated hypoglossal nerve palsy caused by a high cervical osteophyte. This case increases clinical knowledge of an underreported condition and teaches the clinician radiologic pearls in making the diagnosis. To their knowledge, this is the first case report showing surgical remediation of this condition. *Symptoms and Clinical Findings*. A 73-year-old female presented with several months of occipital headache, progressive dysarthria, dysphagia, and tongue deviation to the right. Her neurologic exam was significant for atrophy of the right hemitongue with tongue fasciculations. On protrusion, her tongue deviated rightward. *Diagnosis and Therapeutic Intervention*. Careful review of her initial head computed tomography (CT) imaging revealed that a high cervical osteophyte caused unilateral, isolated hypoglossal nerve palsy. Neurosurgery performed a right, far lateral approach for decompression of this osteophyte and over the ensuing months her symptoms improved.

**Conclusion:**

High cervical osteophyte is an underrecognized cause of isolated hypoglossal nerve palsy. The imaging investigation should be systematic and focus on the skull base with magnetic resonance imaging (MRI) or CT. This is a rare occasion when high resolution CT of the skull base can actually be the more helpful imaging modality. As shown in this case, an osteoarthritic cause can be surgically ameliorated.

## 1. Case

A 73-year-old woman with a history of osteoarthritis and hypertension complained of a chronic but progressive right occipital headache and was developing problems with her tongue. Six weeks prior to presentation, she noticed uncoordinated tongue movements, tongue deviating right on protrusion, and dysarthria. She had difficulty manipulating a food bolus in her mouth and continually bit her right cheek and tongue. Subsequently she had lost ten pounds. She denied facial weakness, ptosis, diplopia, facial sensory changes, or taste perversion.

Her neurologic exam was significant for atrophy of the right hemitongue with tongue fasciculations. On protrusion, her tongue deviated rightward. Taste was preserved. The remainder of her cranial nerve, facial motor, and peripheral motor exam, including muscle bulk and tone were normal. There were no peripheral fasciculations. Sensory exam and reflexes were normal.

Without detailed history of tongue weakness, an initial noncontrast head CT was read as normal. MRI brain with and without contrast showed atrophy of the right tongue associated with subtle fatty infiltration and T2 hyperintensity suggestive of denervation atrophy. Lumbar puncture and erythrocyte sedimentation rate were normal. Electromyography (EMG) with nerve conduction study was performed to evaluate for more diffuse neuromuscular disease. The only abnormal finding on EMG was a subacute right hypoglossal neuropathy.

**Figure 1:**
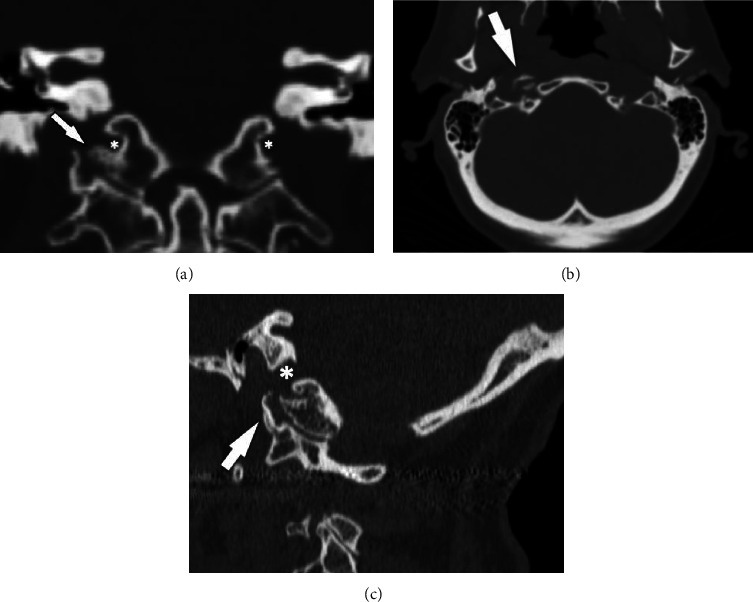
(a) Coronal reformatted CT image shows osteophytosis (arrow) of the atlanto-occipital articulation on the right. The osteophyte arising from the occipital condyle narrows the hypoglossal canal (asterisks). (b) Axial CT showing osteophyte in the right hypoglossal canal. (c) Sagittal CT showing osteophyte in the right hypoglossal canal.

**Figure 2:**
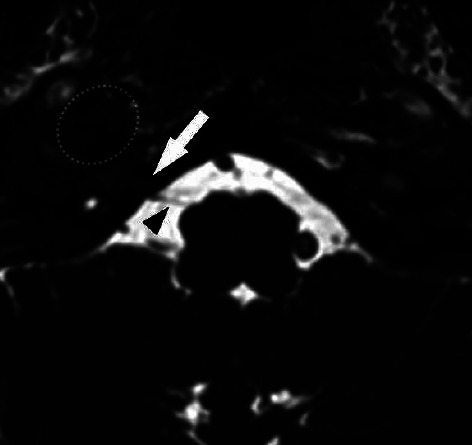
Axial steady-state free procession MR image through the skull base shows the hypoglossal nerve (arrowhead) passing through the hypoglossal canal (arrow). The osteophyte (dashed oval) lies just anterior to the hypoglossal canal, along the expected path of the hypoglossal nerve.

After reviewing the head CT and MRI with neuroradiology and including clear clinical information, degenerative osteophytes were seen projecting from the right atlanto-occipital joint laterally to the adjacent right carotid space, best seen on the CT (Figures 1(a)–1(c)). The osteophyte could be seen at the exit of the right hypoglossal canal on axial MRI images but it was much more difficult to visualize (Figure 2).

Based on our patient's workup, we concluded that she had an isolated hypoglossal nerve palsy from an atlanto-occipital osteophyte compressing the nerve as it exited the hypoglossal canal. Neurosurgery was consulted. To decompress the nerve and stabilize her condition, surgery was indicated.

**Figure 3:**
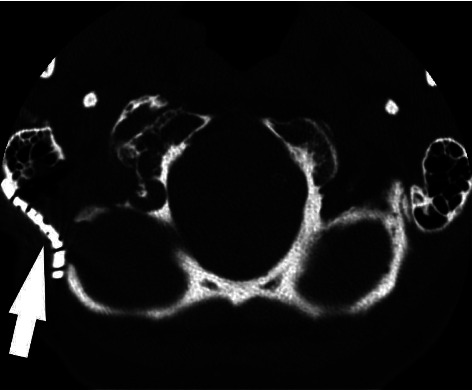
Axial CT image showing postoperative appearance. The hypoglossal canal was decompressed laterally via partial mastoidectomy, high cervical dissection, and partial resection of the occipital condyle. Metallic plating (arrow) covers the postoperative site.

The patient was taken to the operating room for a right, far lateral approach for decompression of this osteophyte. Subperiosteal dissection was used to expose the suboccipital bone and the mastoid bone. The high-speed drill was used to turn a suboccipital craniotomy and perform a partial mastoidectomy to decompress the sigmoid sinus and the jugular bulb. The high cervical region was then dissected. The occipital and C1 condyles were exposed and the drill was used to resect the condyle down to the level of the hypoglossal canal. This decompressed the hypoglossal nerve adequately without having to remove the osteophyte. The dura was then closed in a water tight fashion. The bone was replaced using the plating system. The muscles were reapproximated. The scalp was then closed in layers. Postoperative CT is shown in Figure 3.

She noticed improvement within months that has persisted over 3 years. Her tongue function including eating and swallowing was much improved. The right half of her tongue was less atrophic. The tongue now protruded midline. Her right occipital headache had resolved. Only some mild numbness around her surgical scar remained. From her perspective, she was pleased with the surgical outcome. To the best of our knowledge, this is the first described surgically ameliorated case of hypoglossal nerve palsy caused by high cervical osteophyte.

## 2. Discussion

Hypoglossal nerve injury is not uncommon when it occurs with other lower cranial nerve palsies [1], but isolated hypoglossal nerve palsy is rare. Isolated hypoglossal nerve palsy has been previously reviewed [2, 3]. The best way to ascertain the cause of hypoglossal nerve injury is to use an anatomical segmental approach. By subdividing the course of the hypoglossal nerve into five sections (brain stem, premedullary cistern, skull base/hypoglossal canal, nasopharyngeal/carotid area, and sublingual area), the number of differential causes in each area becomes more manageable and easier for the clinician to remember [3].

The most common cause of isolated hypoglossal nerve palsy is skull base tumor (either metastasis or primitive malignant or benign tumor), accounting for up to 50% of cases [2, 3]. Cranial trauma producing occipital condyle fracture is the second most common cause. This cranial trauma tends to occur in two ways—deceleration trauma from road accidents [4] or surgical trauma. The most common surgical injury is neck surgery or prolonged cervical hyperextension during anesthesia [3]. The third most common cause of isolated hypoglossal injury is internal carotid artery dissection. Cervical rheumatoid arthritis and cervical osteophytes from osteoarthritis have been more rarely implicated [5–7]. In children and adolescents, infectious mononucleosis should be investigated [2].

More modern case series are questioning if hypoglossal nerve palsy caused by craniocervical junction (CCJ) degenerative disease is an under-recognized entity because of provider unawareness compounded by difficulty in identifying the offending lesions on initial CT or MRI standard formats [7]. This was highlighted by our case. Alerting the radiologist to one's suspicion of this diagnosis is critical so they can dedicate attention to the skull base and perform CT reconstructions if necessary. A retrospective series from the Mayo Clinic of 18 patients with hypoglossal nerve palsy from CCJ degenerative disease concluded that a small field of view; thin-section (≤2 mm) axial, coronal, and sagittal soft tissue; and bone reconstructions centered at the hypoglossal canal are more effective at identifying the offending CCJ degenerative disease [7].

The imaging investigation should be systematic and focus on the skull base with MRI or CT [1]. This is one of those rare occasions when high resolution CT of the skull base can actually be the more helpful imaging modality. This was the case with our patient. As shown in our case, an osteoarthritic cause can be surgically ameliorated. The patient was pleased at the improvement in most neurologic symptoms and resolution of her right occipital headache. Since the osteophyte was left in place postoperatively, the headache was likely due to associated CCJ degenerative disease and improved after the surgery. Retrospective case series of 18 patients with hypoglossal nerve palsy from CCJ degenerative disease identified major associated complaint of new or worsening headache or suboccipital pain in 13 of 18 patients [7]. Her timely presentation and surgery within two months of symptom onset likely contributed to the favorable outcome.

## Data Availability

The clinical data used to support the findings of this study are included within the article.
